# Nano-Drug Delivery Systems Entrapping Natural Bioactive Compounds for Cancer: Recent Progress and Future Challenges

**DOI:** 10.3389/fonc.2022.867655

**Published:** 2022-03-29

**Authors:** Vivek P. Chavda, Aayushi B. Patel, Kavya J. Mistry, Suresh F. Suthar, Zhuo-Xun Wu, Zhe-Sheng Chen, Kaijian Hou

**Affiliations:** ^1^ Department of Pharmaceutics and Pharmaceutical Technology, L.M. College of Pharmacy, Ahmedabad, India; ^2^ Pharmacy Section, L.M. College of Pharmacy, Ahmedabad, India; ^3^ Department of Pharmaceutical Science, College of Pharmacy and Health Sciences, St. John’s University, New York, NY, United States; ^4^ Department of Preventive Medicine，Shantou University Medical College, Shantou, China; ^5^ Department of Endocrine and Metabolic Diseases, Longhu Hospital, The First Afliated Hospital of Shantou University Medical College, Shantou, China

**Keywords:** cancer, chemotherapy, drug delivery system, natural bioactive compound, phytochemical, nanomedicine

## Abstract

Cancer is a prominent cause of mortality globally, and it becomes fatal and incurable if it is delayed in diagnosis. Chemotherapy is a type of treatment that is used to eliminate, diminish, or restrict tumor progression. Chemotherapeutic medicines are available in various formulations. Some tumors require just one type of chemotherapy medication, while others may require a combination of surgery and/or radiotherapy. Treatments might last from a few minutes to many hours to several days. Each medication has potential adverse effects associated with it. Researchers have recently become interested in the use of natural bioactive compounds in anticancer therapy. Some phytochemicals have effects on cellular processes and signaling pathways with potential antitumor properties. Beneficial anticancer effects of phytochemicals were observed in both *in vivo* and *in vitro* investigations. Encapsulating natural bioactive compounds in different drug delivery methods may improve their anticancer efficacy. Greater *in vivo* stability and bioavailability, as well as a reduction in undesirable effects and an enhancement in target-specific activity, will increase the effectiveness of bioactive compounds. This review work focuses on a novel drug delivery system that entraps natural bioactive substances. It also provides an idea of the bioavailability of phytochemicals, challenges and limitations of standard cancer therapy. It also encompasses recent patents on nanoparticle formulations containing a natural anti-cancer molecule.

## Highlights

- Traditional medicines hold a significant role in many healthcare systems around the world.
- According to a World Health Organization (WHO) assessment, conventional medicine meets and/or supplements the fundamental health needs of around 80% of the community in underdeveloped nations.
- Plant-derived anticancer medicines are highly sought after because they are effective inhibitors of cancer cells.
- Although the development of nano drugs is fraught with uncertainty, and the development of potent bioactive chemicals from natural origin is not a popular alternative today, improving the effectiveness of known natural bioactive substances using nanotechnology has become a typical aspect.
- Different drug delivery systems entrapping natural bioactive compounds for cancer treatment are summarized.
- Application of Nano drug-delivery systems for natural anticancer agents can improve bioavailability, biodistribution, therapeutic activity, drug targetting, and stability.

## Introduction

Cancer is an abnormal and autonomous cell proliferation caused by a lack of replication control (1, 2). Delayed assessment and non-responsive treatments are the leading reasons for elevated death rates in cancer patients. Knowledge of the principles underlying tumor biology has resulted in substantial advances in cancer control, diagnosis, and therapy in recent years. Surgery, immunotherapy, radiation therapy, chemotherapy, and targeted hormone medications are all traditional ways of cancer management (3). These strategies are often restricted due to their inadequate specificity, since they might also influence healthy cells and the host immune system, resulting in undesirable adverse effects. Furthermore, except for surgery, all medicines employed in cancer management can generate drug resistance in tumor cells. Therefore, anticancer medication research continues for new and better ways to treat tumors. Medication tolerance remains a major issue in cancer care (3). When there is a poor or no reaction to anticancer medication at the start or throughout therapy, resistance might be innate or inherited. Multidrug resistance (MDR) occurs when a patient develops resistance to one treatment and becomes tolerant to additional unrelated drugs (4). Tumors are made of two fundamental components, replicating neoplastic cells and supporting stroma of connective tissues with blood vessels. Mixed tumors, tumors of parenchyma cells, mesenchymal tumors, and tumors of more than one germ cell layer are all different forms of cancer (5, 6). Exogenous carcinogens might be physical, chemical, or biological (7). Biological carcinogens comprise viruses, bacteria, and parasites. Endogenous chemicals of anabolic and catabolic processes, and cells carrying a dormant virus, are the foundation of carcinogenesis (8). Prevention of carcinogen production, reduction of their activation, and enhancement of their elimination can aid in the prevention of cancers (9). Cancer risk factors in humans involve alcohol use, cigarette use, nutritional inadequacy, insufficient physical activity, pollution, and noncommunicable diseases. Chronic diseases including *H. pylori, hepatitis B, hepatitis C, human papillomavirus*, and *Epstein-Barr virus* are also considered risk factors for cancer (10). Diagnosis of cancer is carried out by tissue sample analysis, blood test, Computed Tomography (CT) scan, endoscopy, and many more such methods. Radiotherapy, chemotherapy, and surgery are the most common cancer treatments; however, novel technologies are also underway (11). Most radiation therapy is used for the treatment of glioblastoma, breast cancer, cervical cancer, laryngeal cancer, and others. Radiation includes high-energy ionizing radiation like X-ray and gamma rays which target the tumor cells and damage DNA directly or through the generation of free radicals (12).

Chemotherapy is a term that refers to a medication that involves the use of synthetic chemicals. Anticancer drugs can be used alone or in combination to damage cancer cells or prevent the growth of a tumor. The main issue with chemotherapy is the side effects associated with anticancer drugs. Side effects result from a lack of specificity towards tumor-causing cells. Anticancer drugs can also affect our normal cells, which may lead to side effects (13, 14). Vinca alkaloids, taxanes, epipodophyllotoxins, camptothecins, genistein, and quercetin or rutin are some plant products associated with cancer treatment. Such herbal medications have a lower index of side effects than allopathic medications that are effective in cancer treatment. Some hormonal treatments are also available for the treatment of tumors. Hormones such as glucocorticoids, estrogen, progesterone, and gonadotropin-releasing hormone (GRH) analogs are effective against some cancers. Radioactive isotopes also have a place in chemotherapy, for example, radioactive iodine ^131^I is used to treat thyroid cancer (15). Recent developments of plant-based medicines and research associated with them describe that herbal medications for cancer treatment may replace chemotherapy and can be more effective against tumors (16).

## The Role of Ayurveda in Cancer Therapy

Ayurveda is one of the ancient medicinal plant systems (6000 years old) utilized in the current time for curing or suppressing numerous cancers using natural drugs and their extracts (17, 18). The objective of Ayurveda therapy in cancer treatment is to supplement the mind’s self-healing powers. It aids in the diagnosis of cancers and provides information on herbs and alternative treatments (19). Cancer-like diseases not only affect physical health but also affect mental health. Discordances between the mind and the body cause a variety of symptoms such as lethargy, anxiety, restlessness, and depression (20). When cancer strikes, it causes disorders in the body and affects the *Tamas* (it is the energy that holds all things together over time) and the *Kapha*. Ayurveda offers a significant psychotherapy technique that has been utilized and is currently being implemented in various nations. Plants are an important resource for medication. Plants like vinca, shattering, guduchi, triphala, and tulsi have been utilized as anticancer agents (21). Ayurveda’s goal in treating cancer is to provide preventive, curative, therapeutic, and prophylactic care. Cachexia is a tumor-induced metabolic alteration that triggers an immune response, for which several ayurvedic herbs are used. Ayurvedic medications may not only promote full recovery but also minimize adverse effects in cancer therapy (20). The first goal of Ayurvedic cancer therapy is to regulate and manage *Tridosha* [Vata (wind), Pitta (bile), and Kapha (phlegm), corresponding to the three elements of the universe: air, fire, and water] and *Triguna* (Sattva, Rajas, and Tamas - integral components of the mind). As per Charaka and Sushruta Samhitas, cancer can be classified as *Arbuda* (major neoplasm), and *Granthi* cancers (minor neoplasm) which fall under group 1 cancer as per Ayurveda that includes *Raktarbuda* (leukemia), *Mamsarbuda* (melanoma), and *Mukharbuda* (oral tumor) (22, 23). Group 2 cancer comprises tumors that are *Tridosaj Gulmas* (tumor of stomach and liver). Group 3 diseases include *Asadhya Kamala* (jaundice) and *Nadi Varna* (sinusitis) (22, 24).

**Figure 1:**
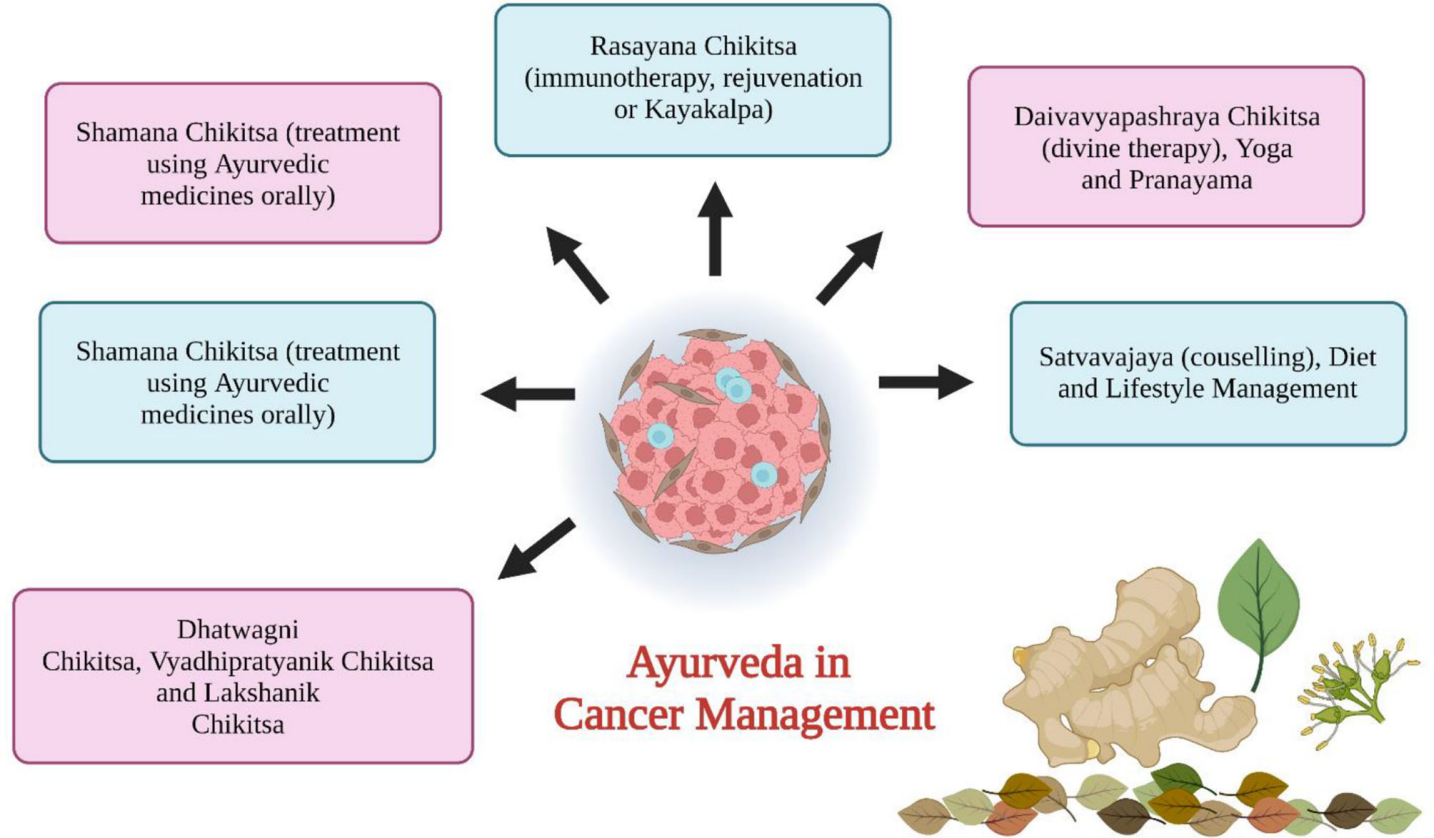
Different cancer management procedures in Ayurveda.

Triguna has an important role in health research fields which contains guna (energy), sattva (quality of reality and purity), rajas (enthusiasm), and tamas (quality of dullness) (25). Triguna is available to everyone and variations in that indicate the health status and nature of a person (26). Ayurvedic treatment of cancer (**Figure 1**) involves i) cleaning and removal of doshas (Sodhna Chikitsa using Panchkarma), ii) serenity of doshas (Somana Chikitsa), iii) rectification of defects (Dhatwangi Chikitsa), iv) immunotherapy (Rasayana Proyaga), v) antineoplastic drugs (Vyadhipratyanika Chikitsa), vi) symptomatic therapy (Lakshanika Chikitsa), vii) surgery (Sastra Chikitsa), viii) maintenance of patient health (Prakritisthapani Chikitsa), ix) restoring the body from disease condition (Rognashini Chikitsa), and x) spiritual treatment (Naishthiki Chikitsa) (26, 27). The general ayurvedic treatment includes maintenance of healthy lifestyle, maintenance of fit digestive power, removal of toxins from the body by *Panchkarma* (method of cleansing the body of all the unwanted waste after lubricating it), revivification by use of Rasayana. Ayurvedic treatment can also be used along with chemotherapy or radiotherapy (28).

## Herbal Plants Exhibiting Anticancer Activities

Human bodies are made of millions of cells, each of which is a self-contained living organism in and of itself. Ordinary body cells proliferate and replicate for a short amount of time before ceasing to do so. Afterward, they only proliferate when they need to rebuild damaged or dead cells. When this cellular replication mechanism becomes uncontrollable, a tumor develops. Cancer cells’ aberrant expansion and division are triggered by DNA destruction in these cells (20).

Traditional medicines hold a significant role in many healthcare systems around the world. Plants’ medical and economic advantages are being more widely recognized and developed in both developing and developed countries. A plant or plant component utilized for its aroma, flavor, and/or medicinal characteristics is known as the herb. Herbal medications and phytomedicines are all terms for products manufactured from herbs that are used to preserve or promote wellbeing (29, 30). Plant-based medications make up around a quarter of the current Indian pharmacopeia, according to estimates. Conventional medicinal herbs are organically existing plant-derived drugs that have been utilized to alleviate disease in local or regional healing traditions with little or no chemical modification (31). Preventive care, disease control, serious adverse effects associated with synthetic medicines, and inadequate therapeutic choices for critical diseases are some of the factors for using herbal pharmaceuticals (22, 30). Tibetan traditional medicine is still somewhat localized in their nation of origin, while others, like Ayurvedic and Chinese traditional medicines, are becoming more widely utilized across the world. Plants filled with chemicals that may have chemoprotective properties are undergoing clinical studies (32). **Table 1** summarized and describes several plant species based on their common mechanism of action and anticancer therapeutic activities.

**Table 1:** Herbal plants with anticancer properties.

Plant name	Part of plant	Family	Phytochemicals	Therapeutic anticancer action	Mechanism of action
*Achillea wilhelmsii k.* (Yarrow) (33)	Leaf essence	Asteraceae	Methanol, flavonoids, 1,8-cineole and α-piene	Breast, colon, and stomach cancer treatment	Suppress reproduction of cancer cells through inducing apoptosis
*Aconitum napellus L.* (Aconite) (29, 34)	Dried root	Ranunculaceae	Aconitine, hypaconitine, neopelline, napelli neoline	Treatment of rheumatism, inflammation, and melanoma	inhibition of Phosphatidylinositol 3-kinase (PI3K)/protein kinase B (AKT) signaling pathways Mitogen-activated protein kinase (MAPK)/extracellular signal-regulated kinase (ERK)1/2 signaling pathways
*Acronychia Baueri schott* (Aspen) (29, 35)	Bark and leaf extract	Rutaceae	Triterpene lupeol and alkaloids-melicopine, acronycin, and normelicopidine.	Antitumor activity in adenocarcinoma and leukemia	Cytotoxic-causes cell death
*Allium sativum L.* (Garlic) (29, 36–39)	Fresh garlic extract, aged garlic, garlic oil, and several organo sulfur compounds	Liliaceae	Methyl allyl trisulfide, diallyl trisulfide, allicin, s-allyl cysteine, s-allyl mercapto-L-cysteine	Anticancer activity in breast cancer cell lines	Cell cycle arrest Generating reactive oxygen species (ROS), Activate stress kinases, Stimulates the mitochondrial pathway for apoptosis Cyclooxygenase 2 (COX-2) suppression, Caspase-3 activation
*Ammi majus L.* (Bishop’s weed, bullwort) (1, 40, 41)	White flower	Apiaceae	Psoralen	Anticancer effect on MCF7 and HeLa cell line	Inhibit cytochrome p450 activity
*Amomum tsaoko* (Chinese black cardamom) (42, 43)	Essential oil, leaf, and seed extracts	Zingiberaceae	18-cineole, geraniol, geranial, α-terpineol, α-phellandrene, Neral, β-pinene, p-propyl benzaldehyde	Antiproliferative action in liver ovarian, and cervical cancer. For adenocarcinoma treatment.	Suppression of signal transducer Activator of transcription 3 (p-STAT3)/nuclear factor kappa-light-chain-enhancer of activated B cells (NF-kB)/interleukin 6 (IL-6) and vascular endothelial growth factor (VEGF) loop
*Aniba rosaeodora ducke* (Pau-rosa) (44)	Wood oil	Lauraceae	Essential oil, linalool	Cytotoxic activity in skin cancer	Depolarization of the mitochondrial membrane Caspase-dependent cell death characterized by phosphatidyl serine externalization
*Artemisia absinthium L* (Wormwood, absinthium) (30, 45)	Plant extract	Asteraceae	Quercetin, isorhamnetin, alphapinin, kamfrolinalol, limonene, myrcene, α-pinene, β-pinene, limonene, artemisinin, artesunate	Anticancer activity in leukemia, colon cancer, breast cancer, hepatic cancer, and melanoma	Inhibiting cell’s growth Apoptosis Preventing angiogenesis Preventing cell migration, Decreasing responses of core receptors
*Artemisia capillaries thunb* (Wormwood) (46, 47)	Unexpanded flower heads	Asteraceae	Essential oil, aantonin, artemisinin	Antioxidant, Anticancer effect in leukemia, prostate cancer, lung cancer, liver cancer, and breast cancer cell lines.	Inhibiting cell growth and induction of apoptosis, Reduction in expression of proliferating cell nuclear antigen (PCNA), Inhibiting the PI3K/AKT pathway
*Astragalus membranaceus Bunge* (Milkvetch) (29, 48, 49)	The root	Fabaceae	Polysaccharides, Flavonoids, and Saponins	Antitumor, immuno modulating, antioxidant, and anti-inflammatory	Direct antiproliferation or pro-apoptosis effect on tumor cells
*Astrodaucus Orientalis L (* 1, 50, 51 *).*	Extract of root and above-ground plant parts	Umbellate	α-Pinene, α-thujene, α-copaene, fenchyl-acetate, anisole, myrcene, and sabinene	Anti –proliferation effects on breast cancer cells (T47D)	Inhibits cell cycle and also induction of apoptosis
*Beta vulgaris L.*(Beet) (52)	Root extract	Amaranthaceae	Betalains, betacyanins, and feruloylbetanin	Anticancer effect in breast cancer, and colorectal cancer	Cytotoxic action
*Boswellia sacra fluck* (Arabian incense) (53, 54)	Resinous dried sap, and essential oil	Burseraceae	α-pinene, α-thujene, β-pinene, myrcene, boswellic acid, α-phellandrene	Antiproliferative, and anticancer action in breast and bladder carcinoma	Inhibiting tumor growth Induces apoptosis with severe damage to cells by activating caspases
*Camellia sinensis (L.)kuntze* (Green tea) (55, 56)	Prepared leaves and leaf buds	Theaceae	(+)-gallocatechin (–),-epicatechin (–),-epigallocatechin (–),-epicatechin gallate, epigallocatechin gallate	Antimutagenic, and antibacterial	Inhibits 5-alfardoctase enzyme in prostate cancer
*Camptotheca Acuminate decne* (29, 57)	Bark seeds, and dried stem wood	Nyssaceae	Camptothecin, quinoline alkaloid (camptothecin, and 10- hydroxy camptothecin)	Antileukemia	DNA–topoisomerase inhibitors
*Casearia sylvestris Sw.* (Wild sage) (58)	Shrub(wild)-leaf extract, essential oil, bark, seed oil, and macerated roots	Salicaceae	α-pinene, α-humulene, β- caryophyllene, bicyclogermacrene, spathulenol	Cytotoxic, and antitumor action	Cell proliferation inhibition
*Catharanthus roseus (L.).G.Don.* (Vinca, periwinkle) (59)	Dried whole plant	Apocynaceae	Vinca alkaloids (Vincristine, vinblastine, leurosine, vindesine, and vinorelbine), ajmalicine, catharanthine, vindoline	Anticancer, and antineoplastic	Cell cycle arrest by inhibition of spindle formation
*Citrullus colocynthis(L.) Schrad* (Bitter apple) (60, 61)	Yellow bitter fruit	Cucurbitaceae	Quercetin, β-sitosterol, and cucurbitales	Anticancer effect in liver cancer, breast cancer, and larynx cancer	Inhibition of cell cycle and apoptosis induction
*Commiphora gileadensis (L.)C.Chr* (Balsam of Gilead) (62)	Gum, fruit, and essential oil	Burseraceae	Sabinene, germacrene-D, α-pinene, β-caryophyllene	Antiproliferative in skin cancer	Increase in caspase 3 activity
*Crocus sativus L.* (Saffron) (63, 64)	Stigma	Iridaceae	Crocin, crocetin, picrocrocin, and safranal	Anticancer in cervical and breast cancer	Inhibiting DNA synthesis
*Curcuma longa L.* (Turmeric) (65)	Dried as well as fresh rhizome	Zingiberaceae	Curcumin, and curcuminoids	Antitumor activity in cervical cancer, leukemia, and lymphoma	Inhibition of telomerase activity
*Curcuma zedoaria Salisb* (White turmeric, zedoaria or gajutsu) (29, 66, 67)	Rhizome	Zingiberaceae	Isocurcumenol, α-curcumene	Antitumor activity in ovarian cancer, cervix cancer, and uterine cancer	Inhibiting the proliferation of cancer cells without inducing significant toxicity to the normal cells. Chromatin condensation, DNA cleavage, nuclear fragmentation, and activation of caspases
*Daucus carota L.* (Wild carrot, birds nest, Queen Anne’s lace, Devils Plague, Bee’s nest Plant) (68)	Seed, root, leave, and flower	Apiaceae (Umbelliferae)	Epilaserine	Inhibitory effect on leukemia cells, breast cancer, and colon cancer	Down-regulation of ERK
*Ferula assa-foetida* L. (Asafoe tida) (69)	Resin	Apiaceae	Coumarin compounds, β-sitosterol and oleic acid.	Antitumor activity in colon cancer, liver cancer, ovarian cancer, and lung cancer	Impairing gene mutation, Affecting enzyme function, Preventing DNA degradation, Influencing cell proliferation, and Altering enzyme action in the cells.
*Glycyrrhiza glabra L.* (Licorice) (70)	Root and stolon	Leguminosae	Licochalcone, glycurrhizin, and glycyrrhizinic acid	Antitumor activity in gastric cancer	Arrests cells in G2/M were accompanied by suppression of cyclin B1 and CDC2. Phosphorylation of Rb inhibited Expression of transcription factor E2F decreased along with the reduction of cyclin D1 Down-regulation of cycline dependent kinase (CDK) 4 and 6 along with increased cyclin E expression
*Hydrastis Canadensis L.* (Golden seal) (71, 72)	Rhizome	Ranunculaceae	Hydrastine, berberine, berberastine, hydrastinine, tetrahydroberberastine, and canadine	Anticancer activity in liver, lung cancer, colon cancer, and oral cancer	Inhibits serine/threonine-protein kinase (PAK) 4 activity and its signaling pathways
*Inonotus obliquus (fungus)* (Chaga mushroom) (73)	Leaves(wild)	Hymenochaetaceae	3β-hydroxy-lanosta-8, 24-dien-21-al, inotodiol and lanosterol	Anticancer-(lung carcinoma A-549 cells, stomach adenocarcinoma AGS cells, breast adenocarcinoma MCF-7 cells, and cervical adenocarcinoma HeLa cells)	Stimulate the immune system, Promote apoptosis, and inhibit angiogenesis
*Lagenaria siceraria(molina) Standl* (Bottle gourd) (74)	Aerial parts	Cucurbitaceae	Vitamin C, β-carotene, vitamin group B, saponins, and cucurbitacin	Breast cancer, and lung cancer treatment	Cytotoxic to cells
*Larrea divaricate Cav* (Chaparral) (75)	Aqueous extract	Zygophyllaceae	Nordihydroguaiaretic acid, guaiaretic acid and its derivatives	Antiproliferative in breast cancer	Cytotoxic effect in arresting cell viability
*Lepidium sativum L.* (Watercress, Rashad) (1, 76)	Seeds, and aerial part	Brassicaceae	Antioxidants-vitamins E, C, B, A, isotiosinat, and omega-3 fatty acids	Anticancer activity in leukemia, and bladder cancer	Induction of apoptosis, and Antioxidant action
*Lippia alba (mill).N.E.Br* (Bushy mat grass, bushy lippia, hierba Negra and pitiona) (77)	Essential oil	Verbenaceae	Geranial,neral,geraniol,trans-β-caryophyllene,6-methyl-5-hepten-2-one,limonene,linalool	Citral dependent toxicity	Cytotoxicity resulting in cell cycle arrest and induction of apoptosis
*Lycopersicum esculentum mill.* (Tomato) (78)	Leaves	Solanaceae	Phenolics, lycopene, glycoalkaloids, anthocyanin, ascorbic acid, tomatine, and carotenoids	Anti-cancer activity in breast cancer, and prostate cancer	Inhibition of PI3K/AKT signaling pathway
*Medicago sativa L.* (Alfalfa) (79, 80)	Plant extracts	Fabaceae	Phytoestrogens and trepans	Hormone-dependent cancer treatment	Phytoestrogens-strong estrogenic activity of this plant is useful in treating hormone-dependent cancers.
*Morus alba L.* (White mulberry) (81)	Fruit, leaves, root, and bark	Moraceae	Kuwanon G, moracin M, steppogenin-4′-O-β-D-glucoside and mulberroside A.	Anticancer effect in lung cancer patients, and colorectal cancer	Suppressing inducible nitric oxide synthase (iNOS), Inhibit NF-κB activation, and cyclin D1 downregulation
*Myrtus communis L.* (Mort) (82)	Plant extracts	Myrtaceae	Polyphenols, myrtucommulone, and semi-myrtucommulone	Breast cancer treatment	Cytotoxic effect on cell layer with cell apoptosis induction
*Nigella sativa L.* (Fennel flower) (83)	Black seed	Ranunculaceae	Kvyynvny compounds and dinitro-quinone	Anticancer activity in kidney cancer, colorectal cancer, and breast cancer	Induction of apoptosis, and Increased cell morphological changes
*Olea Europe L.* (Olive) (84)	Oil, and leaf	Oleaceae	Pinoresinol, oleuropein, maslinic acid and oleanolic acid	Anticancer activity in colon cancer, and breast cancer	Inhibit cell proliferation and angiogenesis. Breast cancer directly act on her-2 gene
*Panax ginseng C.A.Mey* (Ginseng) (85, 86)	Dried root	Araliaceae	Ginsenoside Rp1 panaxosides, chikusetsusaponin	Anticancer effect in breast cancer	Natural killer (NK) cell activation
*Pfaffia paniculata (martius) kuntze* (Brazilian ginseng) (86)	Roots	Amaranthaceae	Butanolic extract	Anticancer effect in breast cancer	Degeneration of cytoplasmic elements, Significant morphological and nuclear modifications of cancer cells.
*Platycodon grandiflorum (jacq.)A.DC.* (Balloon flower, Chinese bellflower, or platy codon) (87)	Roots	Campanulaceae	Platycodin D (PD) and platycodin D3	Anticancer effect in lung cancer, and skin cancer patients	Inducing apoptosis Up-regulation of Fas/FasL, Mitochondrial dysfunction, Bcl-2 family protein modulation, ROS generation, Inhibition of inhibitors of apoptosis, Mitotic arrest induction, Activation of Mitogen-activated protein kinase (MAPK) pathway, Telomerase activity activation and pro-survival pathways suppression -such as AKT, cell cycle arrest, autophagy, and inhibiting angiogenesis
*Pelargonium peltatum* L. (Mayapple, American mandrake, wild mandrake, and ground lemon) (29, 88)	Dried rhizomes and root of Podophyllum peltatum	Berberidaceae	Podophyllotoxin-resin, podophyllin	Lymphadenopathy, and treatment of certain tumors.	Block cells in the late S to G2 of the cell cycle, DNA synthesis inhibition, Cell cycle arrest
*Polygonum multiflorum Thunb.* (He shouwu, Fo-ti) (89)	Root	Polygonaceae	Anthraquinones, physcion, emodin, and questin	Anti-cancer activity in colon cancer	Inhibition of the enzymatic activity of Cdc25B phosphatase
*Prunus armeniaca L.*(Apricot) (90, 91)	Wood, kerne, seed	Rosaceae	Hydrogen cyanide, camphene, benzaldehyde, hexanol, γ-butyrolactone, γ-terpinene	Anticancer effect in lung cancer, and breast cancer patients	Apoptosis induction, Reduction in the expression level of Bax and c-FLIP genes
*Ricinus communis L.* (Castor seed) (92)	Seeds	Euphorbiaceae	Alkaloids, ricinoleic acid, stearic, linoleic, palmitic acid	Treatment of skin cancer	Cytotoxic and apoptosis induction
*Toxicodendron vernicifluaam* (*Stokes) F.A.Barkley* (Chinese lacquer tree) (93–95)	Bark extract	Anacardiaceae	Gallic acid, fustin, fisetin, quercetin, butein, and sulfuretin	Anticancer effect in lung cancer, pancreatic cancer, breast cancer, colorectal cancer, and uterine cancer patients	Modulation of 5’ adenosine monophosphate-activated protein kinase (AMPK) pathway
*Sanguinaria Canadensis L.* (Bloodroot, Bloodwort, Coon Root) (96)	Rhizome	Papaveraceae	Sanguinarine, chelerythrine	Anticancer potential for prostate carcinoma	Modulation of cyclin kinase inhibitor-cyclin-cyclin-dependent kinase machinery
*Silybum marianum L.* (Cardus marianus, M*ilk thistle*, Blessed milk thistle, Marian thistle) (1, 97)	Seeds	Asteraceae	Silymarin	Anticancer activity	Cell cycle arrest and apoptosis
*Solanum nigrum L.* (Barley and wheat) (98, 99)	Juice of root, fruit	Solanaceae	Lunasin	Cancer-breast cancer treatment, chemopreventive, tonic, laxative, appetite stimulant, treating asthma, skin disease, whooping cough	Increase in DNA fragmentation
*Stephania tetrandra S.Moore* (Agrimony) (100)	Root and aerial part	Menispermaceae	Tetrandrine, fangchinoline, cepharanthine, dehydrocrebanine, roemerine, *N*-methylcoclaurine	Anticancer activity in gastric cancer	Inducing pro-death apoptosis and autophagy
*Handroanthus impetiginosus (Mart. ex DC.) Mattos (*Lapacho tree, Pink Tabebuia, Deep Pink Tahebuia) (29)	flowers, leaves, and roots	Bignoniaceae	β-lapachone, lapachol, napthoquinone	Chemopreventive for liver cancer	DNA topoisomerase inhibition
*Taxus Brevifolia Nutt* (Western yew) (101)	Dried leaves, bark and root	Taxaceae	Taxane, taxol, cephalomannine, 10- diacetyl baccatin, docetaxel and paclitaxel	Lung carcinoma, gastric and cervical cancers and also carcinomas of head, breast, ovary, skin, neck, prostate and colon	Preventing the de-polymerization of tubulins
*Thymus fallax Fisch. et Mey* (Thyme) (102)	Plant oil	Lamiaceae	Carvacrol,p-cymene, thymol, and γ-terpinene	Antioxidant, and antibacterial	DNA repair modulation
*Thymus vulgaris L.* (Garden thyme) (103, 104)	Leaves	Lamiaceae	Thymol and carvacrol	Treatment of squamous cell carcinoma of the head and neck, breast and colorectal cancer treatment	Cell proliferation inhibition
*Tussilago farfara L.* (Coltsfoot) (95, 105)	Flower bud,Leaves	Compositae	Senkirkin, kaempferol and quercetin glycosides, saponins, ascorbic acid, sesquiterpenoid	Anticancer effect in lung cancer patients, colon cancer, and in brain cancer	Cytotoxic effect
*Uncaria tomentosa (Willd. ex Schult.) DC.* (Cat’s claw) (105)	Bark extract	Rubiaceae	Oxindole alkaloid	Treatment of breast cancer	Cell decrease at the G₂/M phase
*Urtica dioica L* (106)	Aqueous and ethanolic root extract	Urticaceae	Phenolic compounds	Esophageal, and prostate cancer treatment	Antiproliferative,antioxidant action
*Viola Odorata L. (*English violet) (107)	Dried aerial parts	Violaceae	Cycloviolacin O_2_ Essential oil, alkaloid, saponins, glycoside of methyl salicylate.	Expectorant,anti-cancer diaphoretic, antibacterial, antipyretic	Cell death by membrane permeabilization
*Viscum album L.* (European mistletoe) (108)	Plant extract	Santalaceae	Viscum album agglutinin-1	Treatment of murine tumors, Lewis lung carcinoma, colon adenocarcinoma 38 and C3H mammary adenocarcinoma 16/C	Biological response modifying agent Cytotoxic action
*Vitis vinifera L.* (Grapeseed) (109)	Leaves, and fruits	Vitaceae	Proanthocyanidins, Resveratrol (Trans-3,4’,5-Trihydroxy-stilbene)	Antitumor,and antioxidant	Simultaneous effects on signaling pathways related to extracellular growth factors and receptor tyrosine kinases; Formation of multiprotein complexes and effect on cell metabolism
*Zingiber officinale Roscoe* (Ginger) (29, 110)	Rhizomes	Zingiberaceae	Volatile oil, fat, fiber, starch, inorganic material, residual moisture and acrid resinous matter.	Anti-cancer and anti-inflammatory agents.	Inactivating NF-κB through the suppression of the pro-inflammatory tumor necrosis factor (TNF)-alpha

## Bioavailability of Phytochemicals

Food-derived chemicals reach the circulatory system and are transported to certain tissues where they exert physiological properties. Nutritional phytochemicals are metabolized and carried by the gastrointestinal epithelium before entering the circulatory system (111). The complexity of the biological system include:

- Food material variety and human subject (digestion difference in infants, adults, and the elderly, even there is difference between female and male digestion)
- Complex interactions occur between food/chemicals throughout storage, processing, digestion, and absorption that potentially alter health benefits.
- The process pathways such as compound solubility, gut pH, gut microbiota metabolism, penetration through the intestinal wall, efficient efflux mechanism, and metabolic activities in the first pass may alter the bioavailability of phytochemicals (112, 113).

As per Meyskens and Szabo, the majority of studies recognize one nutrient as the potential cause while ignoring the impact of other phytonutrients or biological factors. As a consequence, in clinical investigations for a certain molecule, the entire diet must be considered (114). According to Lipinski’s rule of five, a substance will be more effective if it contains not more than (NMT) 5 hydrogen-bond donors, NMT 10 hydrogen-bond acceptors, a molecular mass of NMT 500 daltons, a partition coefficient (log P-value) of NMT 5, and NMT 10 rotatable bonds (115). The exceptions include polyphenols like curcumin and green tea. The lymphatic system, instead of the circulatory system, provides phenols with better bioavailability. Molecular forms of phenolics, including glycone or aglycone, can affect absorption that results in variations in bioavailability.

Through uptake and efflux channels on the epithelial cell surface, the intestinal epithelium tissue provides a significant impact on bioavailability. P-glycoprotein, breast cancer resistance protein, and multidrug resistance protein 2 are the primary chemopreventive drug transporters (116). The ATP-binding cassette transporter family has the transport proteins that help nutrients, medications, and metabolites get back into the intestinal lumen. Only nanomolar amounts were available in the blood because of migration (117).

Many medicines that were transferred from *in vitro* to *in vivo* studies were rejected because their findings were insufficient. The chemicals were discovered to be unstable in the stomach and to have low bioavailability, which might have contributed to the clinical failure. Higher dosages were used to overcome the low absorption, which resulted in toxicity to numerous organs despite effectiveness (118). Adverse responses were also seen in some trials, including the SELECT (Selenium and Vitamin E Cancer Prevention Trial) in prostate cancer, which was terminated owing to a negligible rise in prostate cancer incidences. A follow-up analysis of the individuals revealed a substantial increase in prostate cancer incidence in the high vitamin E group years later, even though they had stopped using the supplements (119).

As a result, assessing the bioavailability of drugs purely based on their physicochemical characteristics becomes challenging. Molecular metabolism changes the bioavailability of the compound. Chemicals that prevent the metabolism of other chemicals can enhance their bioavailability. Piperine (derived from black pepper) inhibits the glucuronidation (metabolism) of some chemicals such as tea polyphenol, epigallocatechin-3-gallate, and curcumin increasing their bioavailability (120). Piperine has also been expected to enhance the length of the intestinal microvilli and the fluidity of the intestinal brush border membrane with an enlarged absorptive surface in the small intestine (121). Quercetin and myricetin can prevent resveratrol sulfation and glucuronidation, resulting in increased resveratrol bioavailability (122, 123).

## Drug Delivery Systems Entrapping Natural Bioactive Compounds for Cancer

Multiple novel drug delivery systems (NDDS) have been established during the last two decades, with the primary goal of improving medication bioavailability, preventing adverse impacts, and preventing drug degradation (124). A drug delivery system is based on the idea that pharmaceuticals should be given directly to the region of activity by the demand of the body, and another delivery system is routed *via* drug delivery into the site of action (125). Additional benefits of NDDS include greater solubility, improved bioavailability, less undesirable side effects, boosted therapeutic action, better stability, and better drug distribution. It also involves the regulation of pharmacokinetics, pharmacodynamics, and immunogenicity (126).

The benefit of NDDS is for more patient convenience in drug administration (126–128). Drugs are released from the drug delivery systems *via* two mechanisms, passive and active targeting. One instance of passive targeting is the preservation of chemotherapeutic drugs in solid tumors, which results in enhanced tumor vascular permeability. Active targeting which includes specific receptors on the surface of the cell and ligand-receptor interactions, is extremely specific for interaction (20, 129).

**Figure 2:**
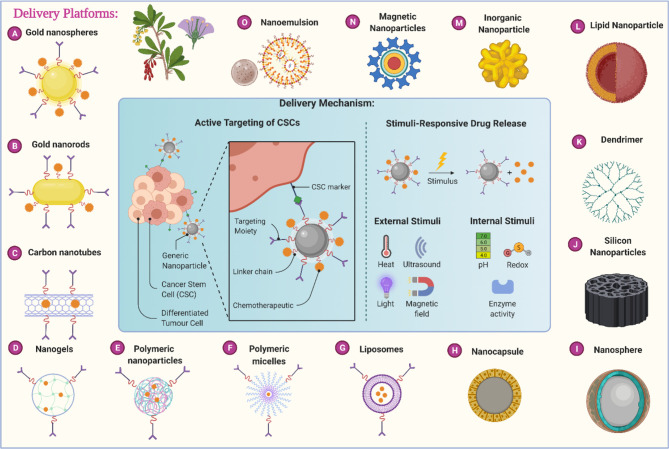
Different nanotechnology-based carrier platforms for the efficient delivery of anticancer phytochemicals and different delivery mechanisms.

Through this review article, we tried to thoroughly evaluate recent statistics on the effectiveness of herbal medicines in cancer with nano-formulations. We have started searching through major databases such as Web of Science, PubMed, Scopus, Elsevier, Springer, and Google Scholar with keywords like “herbs for cancer’ “Nanoherbal formulations for cancer”, “Anticancer Phytochemicals” and then we started with a specific search. From the identified components, first, we have omitted the components for which no strong literature support is available. From the remaining components, we have considered the compounds based on the *in vivo* data evaluation and searched for specific research around anticancer phytochemicals. Later on, from the highly researched components against anticancer phytochemicals, a clinical trial database is evaluated. We have incorporated clinical trial outcomes for the majority of the components if not then in certain cases a cogent discussion on *in vivo* studies is incorporated. Very recent published data is considered for the review of anticancer herbal nanoformulations. NDDS of various sorts are made up of various ingredients, and these drug delivery systems are used for drug administration in the body as well as with certain additional pharmacokinetics and pharmacodynamics properties. **Table 2** illustrates the various drug delivery systems for herbal anti-cancer substances while **Figure 2** summarizes the various nanotechnology-based drug delivery platforms for the delivery of anticancer phytochemicals.

**Table 2:** Types of novel drugs delivery systems for herbal anti-cancer compounds.

Novel Drug Delivery Systems (NDDS)	Phytochemicals	Formulation Components	Remarks	References
Biopolymer-based nanocarrier (BBN)	Curcumin, Quercetin, Resveratrol, etc.	Gelatin, albumin, milk protein, chitosan, pectin, cellulose, guar gum, sodium alginate, starch	Less toxicity, More solubility, More stability, Lower degradation, High biocompatibility	(124, 130)
Liposomes	Carotenoids, epigallocatechin gallate, Curcumin, Quercetin, Resveratrol, etc.	Phospholipids, steroids	High biodistribution, and bioavailability, Increase solubility, Low toxicity	(130–134)
Dendrimers	Curcumin, Paclitaxel, Quercetin, Resveratrol, Ursolic and oleanolic acids, etc.	Polyamidoamine dendrimers (PAMAM), Polypropylene imine dendrimers (PPI), Folate-conjugated polypropylene imine dendrimers (FA-PPI)	Enhanced solubility, Increase drug efflux transporters, Increase bioavailability, Enhanced cell uptake, Low cytotoxicity	(135–137)
Niosomes	Thymoquinone, Curcumin, Quercetin, Resveratrol, etc.	Polyoxyethylene alkyl ethers, Sorbitan monoesters (span 20,40,60 *and* 80), Polyoxyethylene sorbitan monoesters (tween 20, 60, 61 *and* 80)	Lower toxicity, Increase anticancer activity Inhibit P-glycoprotein (P-gp) Better targeted drug delivery	(138, 139)
Polymeric Micelles	Curcumin	Poly(ethylene oxide)(PEO), Poly(ethylene glycol)(PEG), Poly(N-vinyl pyrrolidone)(PVP), Poly(N-isopropyl acrylamide)(pNIPAAm)	Enhanced permeability and retention effect, High stability, Enhanced nutrient and O_2_ demand, Inhibition of efflux pumps to improve the drug accumulation Increase bioavailability of poorly water-soluble drugs Release of the drug in a controlled manner at target sites	(140–142)
Magnetic Nanospheres	Opium alkaloids	Protein, silica, hydroxylapatite, magnetite(Fe_3_O_4_), magnetite (gamma Fe_2_O_3_)	Increase patient compliance, High bioavailability, Reduction of an adverse effect of the drug, Reduces the frequency of dose	(127, 143, 144)
Nanoemulsion	Paclitaxal, rutin, genistein, brucea javanica oil, and coixenolide, etc.	Oil, water, amphiphile, phospholipid, alkyl polyglycosides, PEGylated fatty acid ester, fatty alcohol	Enhanced antigenicity, More potency, Increases humoral response, Enhanced permeability, Improved dispersion of active hydrophobic components and enhanced absorption, Less pain or allergic reaction	(145–149)
Lipid-based Nanoparticles	Curcumin, Quercetin, Resveratrol, etc.	Phospholipids, polyethylene glycol (PEG) PEGylated surfactants	Reduced tumor size, Increase bioavailability to the central nervous system Enhanced Permeability and Retention (EPR) Effect Active drug targeting Stimuli-Responsive and Triggered Release Systems	(127, 150, 151)
Carbon-based Nanoparticles	Tulsi extract, Polyphenols, etc.	Fullerenes, Carbon nanotubes (CNTs), Single-walled carbon nanotubes (SWCNTs), Graphene oxide (GO)	High antimicrobial activity, Inhibition of energy metabolism, Increase of O_2_ uptake, Inhibition of bacterial growth	(152, 153)
Polymeric nanoparticles	Curcumin, Quercetin, Resveratrol, etc.	Chitosan, collagen, Poly(lactic acid) (PLA), Poly(lactic-co-glycolic acid) (PLGA)	Increase accumulation in tumor cells, Stability, Increase therapeutic efficacy Enhanced Permeability and Retention (EPR) Effect Active drug targeting Stimuli-Responsive and Triggered Release Systems	(124, 154)
Nanocrystals	Curcumin, Quercetin, Resveratrol, etc.	Sodium cholate, sodium lauryl sulfate, celluloses, polyvinyl alcohol, hydroxypropylene methylcellulose (HPMC), chitosan, benzalkonium chloride (BAC), hyaluronic acid, polyethylene glycol (PEG), poloxamer	Increase bioavailability, Enhanced transdermal efficacy of poorly soluble drugs, Increases the dissolution rate of drugs, Higher solubility and less tissue irritation Ease of scaling-up	(155, 156)
Nanosphere	Curcumin, Quercetin, Resveratrol, etc.	Polylactic acid(PLA), Polyglycolic acid(PGA), Co-polymer of polylactide-coglycolide (PLGA)	Drug release is delayed, High stability, Increases bioavailability, Increases entrapment of the drug, High antitumor efficiency Optimal dug deposition at the target site	(124, 156)
Nanocapsule	Curcumin, Quercetin, Resveratrol, etc.	Biocompatible hydrophobic polymeric kernel with phospholipid monolayer, and an outer PEG layer	High drug efficiency, Improving poor aqueous solubility, Stabilizing drugs by protecting the molecule from the environment, Providing the desired pharmacokinetic profile, Allowing controlled release, as well as facilitating oral administration	(124, 157)
Metal Nanoparticles	*Tribulus terrestris L.* extract	Gold, silver, iron oxide, copper, zinc oxide, titanium oxide, platinum, selenium, gadolinium, palladium, cerium dioxide	Increase therapeutic action, Enhanced the cellular uptake, Easily combined with drugs, Good biocompatibility, Lower cytotoxicity of drugs, Enhance the sensitivity, Increase in potency, Display antimicrobial activity Radiotherapy enhancement	(158–160)

The drug delivery systems listed in **Table 2** are extensively and separately discussed in the following section of this review.

### Biopolymer-Based Nanocarrier (BBN)

Biopolymers are macromolecules that are divided into three classes: polysaccharides (chitosan, dextran, cyclodextrins, pectin, hyaluronan, guar gum, sodium alginate, cellulose, and starch), proteins (gelatin, albumin, and milk proteins), and nucleic acids (161). To use BBN as a drug delivery vehicle, it is critical to control particle size, the charge on the particle, and specific surface area (161, 162). BBN can provide the high possibility of transporting bioactive compounds to the site of action, which has attracted more attention from chemists, biologists, and pharmaceutical scientists (163). The primary goal of BBN is to elevate the drug’s water solubility, stability, reduce destruction, improve bioavailability, bioactivity, biodegradability, lower toxicity, and flexibility of gel formation (164). Spray drying, electrospray, high–pressure homogenization, supercritical fluid, electrospinning, emulsion–diffusion, reverse micelle, and emulsion – droplet coalescence are just a few of the fabrication methods to engineer BBN (165).

Biopolymer-based biomaterials were studied for use in accelerated diabetic wound healing. Cashew gum (CG) based bio nanocomposite was used as a platform for trials. Crude samples of cashew gum exudate were collected from *Anacardium occidentale L.* for the trial, cashew gum-coated prussian blue nanoparticles (PBNPs) were prepared *in situ* and characterized. CG coating decreased the size of the spherical PBNPs from 50 to 5 nm and the crystallinity of PBNP’s core was increased. The use of CG polysaccharides was found to be an excellent and effective strategy for covering and controlling the size, shape, and crystallinity of prussian blue *via* an *in-situ* synthesis (166). Sebak et al. created noscapine-loaded nanoparticles (NPs) with human serum albumin (HSA) for targeted administration and tested them on SK-BR-3 breast cancer cells using the pH coacervation technique. The results showed that noscapine NPs were substantially more effective than free noscapine in lowering SK-BR-3 cell viability (167). Cationic chitosan (CS)- and anionic sodium alginate (Alg)-coated PLGA NPs loaded with revasterol have been developed by the nanoprecipitation technique (168). For six months, the nanoparticles can inhibit trans isoform breakdown and revasterol escape from the carrier.

### Liposomes

Liposomes are formed from combining cholesterol with phospholipids (128). They shield the enclosed medication from enzyme and other inhibitor activity. They can transport any medication *via* endocytosis and are hence commonly employed for compounds that cannot pass biological membranes. Their disadvantages involve instability, identification by the immune system, and phagocytosis. Conjugation with polyethylene glycol (PEGylation) can shield them from phagocytosis, making them particularly efficient in carrying anticancer medicines (169). Using a thin-film hydration and extrusion method, Lu et al. prepared folic acid conjugated PEGylated liposomes of vincristine for multidrug-resistant cancer therapy and evaluated their cytotoxicity on KBv200 carcinoma cell line and also performed *in vivo* antitumor efficacy studies (tumor growth inhibition and apoptosis assessment studies by TUNEL) (170). The IC50 of the PEGylated folic acid-linked vincristine liposomes was 23.99 nM, compared to 1.10 nM for free vincristine and 363.08 nM for PEG-LS/vincristine. *In vivo* tests revealed that folic acid conjugation greatly improved the antitumor activity of the PEGylated liposomes of vincristine, as well as a higher apoptotic index was captured in the TUNEL assay (24.1 *vs.* 14.4). In another research study, Liposomal ursolic acid showed improved anticancer activity for breast cancer and prostate cancer cell lines than free ursolic acid (171). Recently, a cationic liposome encapsulating hydroxycamptothecin and 5-aminolevulinic acid was administered along with chemo-sonodynamic therapy against metastatic lung cancer that has demonstrated a better apoptotic behavior (172). Moreover, a phase-1 study with 96 non-randomized participants was carried out to assess the effect of liposomal topotecan intravenous injection for the treatment of advanced solid lung tumors. Topotecan is the semi-synthetic derivative of the naturally obtained topoisomerase inhibitor, camptothecin, and the results are yet to be disclosed but the trial is reported to enter phase-2 study by the beginning of the year 2023 (NCT04047251). Inhalable liposomes are being developed and their preparation is studied for use in pulmonary diseases (173). Daunorubicin and cytarabine (vyxeos^®^) were encapsulated in liposomes (Vyxeos^®^, CPX‐351) for the treatment of adults with newly diagnosed, therapy‐related acute myeloid leukemia or acute myeloid leukemia with myelodysplasia related changes (NCT01696084). Vyxeos^®^ is an orphan drug and its clinical trial proved to be remarkable. A clinical study CLTR0310‐301 (also referred to as Study 301) was a phase III, multicenter, open‐label, randomized, trial of vyxeos^®^ (daunorubicin‐cytarabine) liposomal injection versus standard 3 + 7 daunorubicin and cytarabine in patients aged 60–75 years with untreated high‐risk (secondary) (173, 174). A recent clinical trial is investigating the efficacy and safety of paclitaxel liposome as first-line therapy in patients with advanced pancreatic cancer. The patients were given paclitaxel liposome 175 mg/m^2^ intravenously every 3 weeks for 3 weeks until the disease recurrence. All five parameters, including overall response rate, overall survival, disease control rate, quality of life, and adverse events are examined (NCT04217096). Moreover, an open-label, phase-1 study on the use of liposomal irinotecan injection was carried out on 136 participants to study its effects on advanced breast cancer and evaluate its efficacy and safety (NCT04728035). Transfersomes are phosphatidylcholine-containing liposomes with an edge activator (175). Because of greater penetration, celecoxib loaded into transfersomes containing soy phosphatidylcholine combined with sodium deoxycholate was found to be a therapeutically efficient drug-delivery approach for the treatment of rheumatoid arthritis (176).

### Niosomes

Niosomes are obtained through combining an alkyl or dialkyl polyglycerol-based nonionic surfactant and subsequent hydration in an aqueous environment. Because of their comparable construction, they are frequently employed as a replacement for liposomes (132). They are not toxic and restrict the action of drugs to specific locations. Niosomes have also been shown to be manipulable as a part of advanced drug delivery strategies and can deliver drugs *via* intravenous, transdermal, ocular, and pulmonary routes. Cancer therapy, gene therapy, and targeting medicine *via* the nasal route are some of the other uses for niosome based drug delivery. It is also used in immunological applications, such as a hemoglobin transporter, and for cosmetic reasons (177). Morusin-loaded niosomes have been produced for cancer therapy (178). Nanomorusin, unlike free morusin, was shown to be easily dispersible in aqueous environments. Morusin was shown to have an extraordinarily high drug entrapment efficiency (97%), regulated and prolonged release, and increased therapeutic effectiveness in cancer cell lines from four distinct lineages. A different research study evaluated the anticancer activity of niosomes containing both curcumin and methotrexate against colorectal cancer cell lines (179). The niosomal formulation showed more *in vitro* cell toxicity than their combination in free form. The anti-arthritic efficiency of luteolin (LT)-loaded niosomes made with different nonionic surfactants was tested *in vitro* and *in vivo*, with improved entrapment efficacy and higher transdermal flow across the rat skinned discharge. The cytotoxicity study showed a lesser IC50 value from LT-NVs than the pure LT (180). Thus, it can be concluded that LT-NVs are a natural alternative to synthetic drug in the treatment of lung cancer. The therapeutic purpose of niosomes is to deliver anticancer drugs, protein, peptide, and natural product delivery with improved bioavailability. Numerous experts are now intrigued by the delivery of natural compounds such as curcumin, which has low solubility and bioavailability (177). As a result, clinical trials with the combination of *Curcuma longa L.* and doxorubicin are underway (NCT04996004). Aside from curcumin, there are natural compounds called morusin that have powerful antibacterial, anti-inflammatory, and anticancer properties. *Rahman et al.* developed a formulation in which morusin is loaded with niosomes as a carrier, removing the barrier of low solubility and stability to target the specific cell (126). A study to evaluate the efficacy and safety of niosomal docetaxel lipid suspension compared to taxotere^®^ in triple-negative breast cancer patients (after failure to previous chemotherapy) was conducted (NCT03671044). In this randomized, open-label clinical trial, females 18-65 years of age were eligible to apply. The niosomal docetaxel lipid suspension was administered intravenously at three-week intervals at a dose of 75 mg/m^2^. Patients received drug doses until disease progression and unacceptable toxicity occurred. This procedure was repeated with a 100 mg/m^2^ dose of niosomal docetaxel lipid suspension. The trial’s primary goal is to compare the proportion of patients with the best overall response rate in niosomal docetaxel lipid suspension and taxotere^®^ (NCT03671044).

### Polymeric Micelles

Micelles are lipid molecules that organize themselves in a spherical shape in aqueous solutions. They fluctuate in size from 10 to 100 nm and are generally quite narrow in terms of the size distribution (181). They can improve drug absorption and retention by shielding the drug from inactivation by its micellar environment. This system is made up of a core-shell structure with a lipophilic core and a shell made up of hydrophilic polymer blocks (182). The physicochemical properties of the drug and their position in the micelle influence their release. Certain physical stimuli, such as pH, temperature, ultrasound, and light can also promote drug release from the micelles in the targeted location (183). Micelles also provide an increased drug accumulation at the tumor site. Recently, doxorubicin delivery through covalent attachment with the hydrophobic portions of an amphiphilic block copolymer resulted in pH-regulated drug release (184). Paclitaxel, doxorubicin, 5-fluorouracil, 9-nitrocamptothecin, cisplatin, triptorelin, dexamethasone, and xanthone were all microencapsulated in poly lactic-co-glycolic acid (PLGA), polymerized lactic acid (PLA), and polycaprolactone (PCL) nanostructures with a micellar backbone (185). The medications are released to the target. Paclitaxel, for example, is encapsulated in polymeric micelles, which improves the systemic pyruvate kinase (PK) profile and interferes with drug-producing adverse effects such as neurotoxicity. It is USFDA approved and the formulation is marketed as Genexol^®^ PM (183). As a solution to breast cancer and better intracellular delivery of docetaxel, novel pH-triggered biocompatible polymeric micelles have been developed based on heparin–α-tocopherol conjugate. For this, the amphiphilic copolymer was synthesized by grafting α-tocopherol onto the heparin backbone by a pH-cleavable bond (186). The paclitaxel and cisplatin-loaded polymeric micelles in advance non-small cell lung cancer were evaluated as a comparative phase 2 study with 276 participants. The primary endpoint of this study is a response rate of up to 6 cycles to ensure that the overall anticancer response is achieved. The secondary outcome measure is overall survival over three years (NCT01023347). The safety results are satisfactory (data not available in the public domain) and they have started phase 3 studies (NCT02667743).

### Magnetic Microspheres

Magnetic microspheres are made up of microscopic supramolecular particles that are surrounded by capillaries. The primary goal of magnetic microspheres is to deliver drugs to the specific site of action that ultimately reduces the adverse effects. Magnetic microspheres are composed of tiny particles such as protein, silica, hydroxylapatite, magnetite (Fe_3_O_4_), maghemite (gamma Fe_2_O_3_), and synthetic polymers (143). The drug delivery is divided into two parts: spatial placement and temporal delivery of the drug (143). Magnetic microspheres cause pharmaceuticals to be released in a sustained manner rather than having a high bioavailability. Examples of magnetic microspheres-containing products are lupron depot^®^ and nutropin depot^®^ (187). Solvent evaporation, multiple emulsion techniques, phase separation emulsion polymerization, emulsion solvent extraction method, hot melt microencapsulation, and dispersion copolymerization are some methods for producing magnetic microspheres (183). It is used for the treatment of alveolar echinococcosis, a human helminthic disease caused by the larvae of *Echinococcus multilocularis* tapeworms. Treatment of this disease uses iron oxide magnetic particles. Spray-drying was used to create magnetic microspheres, which were then studied for their physicochemical features and dissolution profile. They were also tested for therapeutic effectiveness in both *in vitro* and *in vivo* systems. *In vitro* experiments in B16 melanoma cells found that using 30 M or 50 M sulforaphane with iron oxide in the polymeric carrier inhibited cell survival by around 13% -16%. *In vivo* data from C57BL/6 mice demonstrated that magnetic microspheres (located to the tumor site with the assistance of a powerful magnet) prevented 18% more tumor development than sulforaphane in solution (188). The magnetic microsphere of curcumin and doxorubicin demonstrated potential *in vitro* anticancer activity in cell viability and MTT cytotoxicity studies (189).

### Nanoemulsion

Nanoemulsions (NE) is a colloidal dispersion system, and one of their most essential functions is to act as a tool for increasing the bioavailability of poorly water-soluble medications (147, 148). They are composed of oil, water, amphiphile, phospholipid, alkyl polyglycosides, PEGylated fatty acid ester, and fatty alcohol. They are having mean droplet sizes less than 100 nm. The NE system has prolonged drug release, high solubility, higher skin permeability, more drug absorption, extremely low viscosity, less allergic response, and less drug degradation (190). Oral administration of the medication enhances bioavailability. The hydrophilic substances have higher permeability when administered subcutaneously or intramuscularly, and they are condensed into the lymphatic system (145). Over the last few years, NE has created novel systems such as Transcuto^®^ P, alkyl polyglycosides, etc. Natural herbal medications like rutin, genistein, brucea javanica oil, and coixenolide have been grated into NE for some applications. The antioxidant properties of *Syagrus romanzoffiana (Cham.)Glassman*, fruit O/W (oil in water) NE are assessed using the phase inversion technique (190). Paclitaxel nanoemulsion containing tocopherol as oil phase has been developed and evaluated for targeted cancer therapy that reached phase 3 clinical trials (NCT01620190) (191). The effects of eugenol nanoemulsion on pain were studied *via* a randomized double-blinded controlled cross-over trial (158). For the phonophoretic application of glucosamine and chondroitin in the treatment of knee chondropathies, nanoemulsion (nano CG) has been made and evaluated. Nano CG was previously applied in randomized and controlled clinical trials (192). In this experiment, patients in arm 1 receive nanoemulsion curcumin orally twice daily for up to 3 months. In arm 2, a placebo was administered orally twice daily for three months. This trial’s result was measured using the functional evaluation of cancer therapy (endocrine system inhibitor) to detect changes in aromatase inhibitor. This experiment is currently ongoing, however, it is not currently recruiting participants (NCT03865992). Plumbagin improves the efficacy of androgen deprivation therapy (ADT) in prostate cancer and it is now being evaluated in phase I clinical trial (193). A recent study found that plumbagin decreased PTEN-P2 driven tumor development only in castrated mice but not in intact animals, indicating that dihydrotestosterone (DHT) was generated in the testes and was primarily responsible for inhibiting prostate cell death (194). An oleic-acid-based nanoemulsion of plumbagin increases its antitumor efficacy (193). Nanoemulsion formulation of piplartine resulted in enhanced solubility, oral bioavailability, and anti-tumor efficacy of piplartine (195).

### Nanoparticles (NPs)

NPs are constructed of a range of materials, some of which are poisonous. Biodegradable materials are less harmful. NP systems are being investigated for a wide range of biological applications. NP protects the drug from enzymatic breakdown and allows for regulated drug release (196). Through adhesion to the capillary wall, NPs may improve the oral bioavailability of poorly soluble medicines and tissue absorption following parenteral administration. They may also improve drug transport across membranes. They are vulnerable to phagocytosis and endocytosis because of their hydrophobic surface, which is quickly covered with plasma proteins and taken up through the mononuclear phagocytic system (MPS) present in organs like the liver, spleen, and bone marrow (169).

Applying the ionic gelation process, berberine-loaded NPs were synthesized for anticancer potential (197). The rotary evaporated film ultrasonication process was used to create glycyrrhizic acid-loaded NPs (198). For testing therapeutic synergy in the ulcerative colitis (UC) model, pH-sensitive NPs of the curcumin-celecoxib combination were optimized. Its effectiveness was later validated in a UC model in rats, as per the tests (199). Gold NPs (AuNP) were created using a greener method that included digested *Acorus calamus L.* rhizome as a reductant and chloroauric acid as a starting material. The existence of a surface plasmon resonance (SPR) peak in the ultraviolet (UV)–visible spectral analysis revealed the formation of AuNP (200). Hexagonal antibacterial Zn_0.95_Ag_0.05_O (ZnAgO) NPs have been made using rosemary leaf extracts as a green chemistry method whose formation was confirmed by X-Ray Diffraction (XRD), Fourier transform infrared spectroscopy (FTIR), and UV–visible spectra (201). Clinical trials on magnetic fluid hyperthermia based on magnetic NPs were conducted and studied (NCT01411904). Nanoparticle role in photodynamic therapy for solid tumors has been studied *via* a clinical trial. The aim of the use of nanotechnology-based approaches as delivery tools for photosensitizer (PS) is to study the improvement in their cancer cellular uptake and their toxic properties, as well as the photodynamic therapy’s therapeutic impact (202). Potential anticancer activity is reported for apigenin-loaded PLGA nanoparticles against skin tumor in mice models (203). These nanoparticles showed ameliorative potentials in combating skin cancer and therefore have a greater prospect of use in the therapeutic management of skin cancer. Similarly, significant anticancer activity was reported against osteosarcoma cancer cells (MG63 and Saos-2 osteosarcoma cell lines) by formulating PLGA nanoparticles of etoposide and paclitaxel (204). The results of quercetin-loaded PLGA nanoparticle administration showed potential action against C6 glioma cells (205). It has demonstrated antioxidant properties and cellular oxidative resistance. The results showed that the improved batch (Qu1NPs) exhibited better cellular absorption at a lower IC50 value (29.9 gmL^-1^) after 48 hours of incubation. The NPs may lessen resistance in brain cancers by lowering oxidative stress. A nano-phytocomposite consisting of phytochemical extract (BRM270) has cytotoxic potential against HepG2 human hepatoma cancer cells (206). A randomized, double-blind, placebo-controlled phase 2/3 study is now underway to assess the effectiveness of berberine hydrochloride against the development of new colorectal adenomas in 1,000 people who have a history of colorectal cancer (NCT03281096). A phase-2 trial is investigating the adverse effects of paclitaxel albumin-stabilized nanoparticle formulation for treating diseases such as fallopian tube cancer, primary peritoneal carcinoma, and recurrent ovarian carcinoma. The major goal of the experiment is to assess tumor response after each cycle for the first six months, as well as the frequency and severity of side effects seen. This research is open to people of all ages, however, only women are permitted (NCT00499252).

### Lipid-Based Nanoparticles (Solid Lipid Nanoparticles - SLN)

PEGylated surfactants are used in the production of lipid-based NPs. Lipids act as penetration enhancers of drugs. It also enhances drug solubility and diffusion from the lymphatic to the circulatory system. One of its applications is to improve central nervous system (CNS) bioavailability. They may be manufactured *via* quick, solvent-free, and scalable procedures. Some methods of preparation include nanoprecipitation, emulsification–solvent evaporation (ESE), or high-pressure homogenization (207). The microemulsion technology was used to create curcuminoid solid lipid NPs with the active component being curcuminoids. It has antioxidant and anticancer effects (199). Exocytosis of NPs is the step that details the cytosolic administration of therapeutic agents *via* NP carriers (208). Silymarin-loaded nanostructured lipid carrier (NLC) is the best example that has been used clinically to overcome low solubility, permeability, and bioavailability issues in hepatic diseases (126). Cardamom essential oil (CEO)-loaded NLCs have successfully been synthesized using food-grade lipids which include cocoa butter and olive oil. This modification led to a small size (90%), improved loading capacity (>25%), and provided good physical and chemical stability (126). Paclitaxel-loaded solid lipid nanoparticles showed better anticancer action in the murine breast cancer mice model (209). Several solid lipid nanoparticles have been created for the administration of cytotoxic medications such as doxorubicin, idarubicin, paclitaxel, camptothecan, 7-ethyl-10-hydroxy-20(S)-CPT, etoposide, flurodooxyuridine, and retinoic acid, as well as cholesterylbutyrate (210). The *Ferula assafoetida* seed oil (FSEO) -SLN nanoparticles inhibited the development of the human NT-2 cancer stem cell line significantly (211). They caused apoptosis by increasing the expression of TNF-, P21, and Cas3 genes. The FSEO-SLN inhibited angiogenesis in CAM tissue by reducing the length and quantity of blood vessels. As a result, it has the potential to be investigated as an effective anti-cancer agent. Similarly, Epigallocatechin-3-gallate (EGCG) SLN can improve EGCG bioavailability and stability and can be employed as an alternate approach for EGCG oral delivery (212). In the rat model, SLN-EGCG had neither acute or sub-chronic toxicity when compared to free EGCG.

### Carbon-Based Nanoparticles

Fullerenes, carbon nanotubes (CNTs), single-walled CNTs (SWCNTs), and graphene oxide (GO) are used in the production of carbon-based NPs. They have great antibacterial action and are utilized to limit energy metabolism, reduce or enhance O_2_ absorption, and inhibit bacterial growth (152). They are 3–10 nm in diameter. Their interior structure is made up of graphite sheets that have been glued together to form a quasi-spherical nanoparticle. They are also utilized to make biosensors.

CNTs have been extensively investigated for their potential to deliver anticancer herbal compounds with better therapeutic effectiveness and safety (213). CNTs have been used in both therapeutic and environmental settings. *Ocimum tenuiflorum L.* (tulsi extract) containing photosynthesized silver nanoparticles (AgNP) loaded into emulsified multiwalled carbon nanotube (MWCNT) was developed for fertility analysis targeted to the intracellular part of the sperm cell. It was characterized by a spherical shape, 5–40 nm in size, and surface plasmon resonance imaging (SPR) at 430 nm (126). The total polyphenols content and antioxidant activity of *Echinacea purpurea (L.)Moench.* extracts were determined using glassy carbon electrodes modified with CNTs and chitosan (214). In a recent research study, carbon quantum dots of *Echinops persicus* extract demonstrated efficient antiradical activity (215). The biosynthesized silver nanoparticles of the leaf extract of *Teucrium polium* exhibited significant anticancer activity against the MNK45 human gastric cancer cell line (216).

### Polymeric Nanoparticle and Dendrimers

Polymeric nanoparticles (PNs) are colloidal solid particles that are made up of various polymers such as chitosan, collagen, polylactic acid (PLA), PLGA, or other biodegradable polymers (146). PN enhances the bioavailability of the poorly soluble drugs by allowing the oral administration of the drug and increasing accumulation of the drugs at the site of action since the smaller size of the particle allows penetration into the capillaries and the cells (155). Methods of preparation of polymeric NPs include complex coacervation method, coprecipitation, salting out method, solvent displacement method, and solvent emulsification-diffusion method (185). Solvent evaporation, emulsification/solvent diffusion, nanoprecipitation, emulsification/reverse, and salting-out methods are all used to create nanospheres while nanoprecipitation is used to create nanocapsules. An organic phase is initially generated in the solvent evaporation process, which comprises a polar organic solvent in which the polymer is dissolved and the active component has incorporated either by dissolving or dispersion. Now PNs are approved by FDA for clinical use in the treatment of breast and pancreatic cancer and also some clinical trials are performed in many of the compounds such as ABI-008, ABI-009, and ABI-011(NCT04229004). The free radical process was used to create *A. absinthium* extract-loaded polymeric NPs (NVA-AA), which were efficient against breast cancer cell lines; MCF-7 and MDA MB-231 (217). *Uncaria tomentosa* (Willd. ex Schult.) DC. (UT) extracts have been shown to have promising antitumor activity for the enhancement and delivery, in which PCL and PLGA were employed for the generation of NPs loaded with UT extract by a single emulsion solvent evaporation method (218).

Dendritic packaging of bioactive agents allows for the separation of the active site, which has a structure similar to active sites in biomaterials (137). In addition, unlike most polymers, water-soluble dendrimers may be created by functionalizing their outer shell with charged species or other hydrophilic groups. Capsaicin-loaded dendrimers showed significant cytotoxicity on VERO cell line with an IC50 of 1.25 μg/mL and MCF-7 and HEp2 cell lines with an IC50 of 0.62 μg/mL (219). Polyamidoamine (PAMAM) dendrimers as oral drug delivery carriers for quercetin were evaluated. It was discovered to be in the nanometer range (100 nm) with a low polydispersity index. An *in vitro* investigation indicated a biphasic release pattern of quercetin, with an initial rapid release phase followed by a sustained release phase, and a pharmacodynamic analysis offered early proof of concept for the potential of quercetin-PAMAM complexes (220). Poly (propylene imine) dendrimer is also evaluated for different anticancer phytochemicals (221).

### Nanosphere (NS)

NSs are amorphous colloidal aqueous solutions with sizes ranging from 10 to 200 nm. Because they are tiny in size, they may be easily delivered orally, locally, and systemically, resulting in better bioavailability. They are made up of synthetic polymers such as PLA, polyglycolic acid (PGA), PLGA, and others (222). The main functions of the system include extending drug action due to delayed drug release, boosting bioavailability, enhancing drug entrapment, and boosting drug stability. It can also act against chemical degradation and minimize drug toxicity. Albumin NS, modified NS, starch NS, gelatin NS, polypropylene dextran NS, and polylactic acid NS are all examples of biodegradable NSs (223). Presently, two novel forms of NS have emerged: immune NS and magnetic NS. Both of these NSs are joined to form the immunomagnetic NS, and they considerably enhance drug targeting (222, 224). The targeting ability of the NS is currently being investigated in HeLa cells. Researchers have discovered that the C-phycocyanin (C-pc) NS is employed as a fluorescent producer. The intensity of fluorescence is higher in C-pc, indicating that it was directed at HeLa cells. Studies have revealed that the CD55 specific ligand peptide (CD55sp) is a potent anti-cancer factor. CD55sp is injected into the body of a mouse to assess the distribution of NSs, which are collected in the spleen, liver, and tumor. The liver and spleen metabolize the medication and start phagocytosis against malignancies (225). The pre-clinical trial such as oridonin (ORI)-loaded poly (D, L – lactic acid) (PLA) and polymer (RGD – Gly – Asp peptides) for the better antitumor actions (126). Another study created a near-infrared-responsive pharmacological system based on Au nanocages with Biotin–PEG–SH modification for the combination of doxorubicin and quercetin. The resulting nanocomplex have substantially more effective effects on MCF-7/ADR cell growth suppression under near-infrared irradiation. Furthermore, co-administration of doxorubicin and quercetin might significantly boost doxorubicin intracellular accumulation and dispersion in nuclei (226).

### Nanocapsule

Nanocapsule (NC) is a colloidal dispersion system with a conventional core-shell structure, and the medicine is captured into the cavity of the central regions of the shell, which is surrounded by various polymer components such as polystyrene, titanium oxide (TIO_2_), and silver (Ag). The NC platform has high drug encapsulation efficiency, low polymer content, and a core-shell that protects the medication from degradation elements such as pH and light (227). Nanoprecipitation, arc discharge method, emulsion–diffusion, double emulsification, interfacial polymerization, emulsion–coacervation, polymer coating, and layer–by–layer coating are some of the methods used to create nanocapsules. The layer–by–layer approach is used to improve the hydrophilicity of crystals, such as artemisinin (ART, *Artemisia annua L*) crystals, having good anticancer action. Herbal compounds have been encapsulated with polyelectrolytes for pharmacological self-release (150). There is one other method of preparation known as interfacial polymerization that yields a dispersion of aqueous core nanocapsules (228). Biogenic synthesis of PEG-enhanced *Moringa oleifera Lam* Ag nanocapsules was performed and its antibacterial activity was tested. The Ag nanocapsule (AgNs) formed had a single-phase cubic structure as seen under X-ray spectroscopy (229). Chitosan nanocapsules of tarragon essential oil were made, and had low cytotoxicity and long-lasting activity as a green nano-larvicide. Its cytotoxicity was checked on human skin lines (230). This system is already a clinical trial is completed such as an anti – HER2 mAb with paclitaxel *and* a mAb with doxorubicin (Doxo^®^) (231). Mardani et al. discovered that curcumin nanomicelles might suppress lung metastasis and melanoma cell growth (B16 F10). The time and concentration of incubation are critical elements in imparting the potential activity of nanomicelle. Curcumin nanomicelles cause apoptosis even at low concentrations of 20 M. Curcumin also reduces angiogenesis and controls T Cell activation (232).

### Metallic Nanoparticle

Metallic NPs (MNPs) are comprised of a metal core made of inorganic metal or metal oxide, which is generally surrounded by a shell also made of organic or inorganic material or metal oxide. Gold, silver, iron oxide, copper, zinc oxide, TiO_2_, platinum, selenium, gadolinium, palladium, and cerium dioxide are some of the metals and metal oxides that are employed. Because NPs are poisonous to live cells, their manufacturing and usage in powder form are deemed risky. To address this issue, highly reactive MNPs can be encapsulated (233). Aqueous plant extracts were mixed with metal salt solutions to create MNPs. Secondary plant metabolites are continually involved in the redox process that produces environmentally favorable NPs. The initial indication for MNP production is detected as a color shift. UV visible spectroscopy can be used to track the development of the reaction. Because of the drawbacks of the other approaches, the biological synthesis of NPs is the ideal way for producing these MNPs (234). It is a one-step bio-reduction approach that requires less energy to synthesize eco-friendly NPs. The biochemical reaction of AgNO_3_ reacting with plant broth results in the synthesis of AgNPs, according to the bio-reduction technique. *Tribulus terrestris L* extract was combined with various molar concentrations of silver nitrate solution to create eco-friendly AgNPs with distinct morphological properties. The methanolic extract of the *Callicarpa maingayi Kjng and Gamble* stem is employed in the manufacture of AgNPs and the formation of [Ag (*Callicarpa maingayi*)] + complex. The aldehyde group contained in the extract is mainly involved in the reduction of silver ions into metallic AgNPs (235). *Piper nigrum L.*, leaves were stated to contain an important bioactive compound, involved in the NP synthesis by an eco-friendly method. *Artemisia nilagirica(C.B.Clarke) Pamp.*, leaves were also used for the synthesis of NPs (224). These NPs are used because of their various properties. AgNPs are used as an antibacterial agent, wound dressing material, bone and tooth cement, and for water purification. MNPs are being used in a variety of fields, not simply in medicine and agriculture. MNPs are also employed in the manufacture of several bacteria, including *Klebsiella pneumonia* and *Pseudomonas aeruginosa*. It is also employed in the production of bioactive compounds and phytochemical constituents (236). In now a day the clinical trial is performed in some inorganic nanomaterials such as gold nanoparticles and silica nanoparticles (237). Folic acid surface modification improves the targetability of curcumin magnetic nanoparticles. Curcumin-loaded folate-grafted magnetic nanoparticles inhibit KB nasopharyngeal cancer cells and MCF-7 breast cancer cells significantly. The nanoparticles demonstrated targeted thermo-chemotherapy leading to apoptosis by a selective interaction with folate receptors, which are abundantly expressed in cancer cells, based on magnetic effect (238). The eupatorium-containing mPEG-b-PLGA-coated iron oxide nanoparticles were investigated towards prostate cancer cell lines (DU-145 and LNcaP). Furthermore, it exhibits increased apoptosis and reduced necrosis in the sub G1 phase, as well as enhanced cell populations, rendering it a suitable option for drug-resistant cancer treatment (239). The MTT experiment revealed that eupatorin-loaded Fe3O4@mPEG-b-PLGA nanoparticles significantly slowed the development of DU-145 and LNcaP cells, with IC50 values of 100 M and 75 M, respectively.

### Inorganic Nanoparticle

Inorganic nanoparticles are composed of silver, gold, iron oxide, silica, platinum, and gadolinium hydroxide (Gd(OH)_3_). Magnetic (superparamagnetic iron oxide particles), nanoshell (dielectric silica core in thin gold metal shell), metallic, and ceramic (porous biocompatible substance) nanoparticles are its additional subclassifications based on the material used (240). They have good biocompatibility, can reduce cytotoxicity of medicines, increase the formation of reactive oxygen species (ROS), and have antibacterial action. In comparison to organic materials, these NPs are nontoxic, hydrophilic, biocompatible, and very stable. Inorganic nano-drug delivery methods such as mesoporous silica NPs, CNTs, layered double hydroxides, superparamagnetic iron oxide NPs, and calcium phosphate NPs have been developed as therapeutic uses in a variety of disorders, including neurodegenerative diseases. Magnetic NPs (MN) are employed in magnetic resonance imaging (MRI). Using zirconium oxide NPs, the antifungal activity of neem and aloe vera formulations was sustained and increased (241). In recent years, inorganic NPs have been synthesized and conjugated with tiny drugs for a variety of therapies. Inorganic NPs, which are mostly based on selenium, metallic chalcogenide, silicon, and carbon, are employed in oncotherapy (242). A pre-clinical trial of Anti-EGFR antibody cetuximab (C255) approved by FDA for the treatment of EGFR – positive colorectal cancer and palladium compound (TOOKAD) is a clinical trial by photo-dynamic therapy for the treatment of prostate cancer (65).

## Recent Patents

Experts are currently working to modify particles by combining many newer sorts of compounds. The plant bioactive molecule has been found to provide considerable benefits in cancer therapy; however, administering this component is not easy (243). Consequently, a recent patent provides evidence of a unique herbal ingredient-delivery technique. Although there are still challenges to be resolved, this will become a significant delivery method soon. It has already achieved notable progress in the fields of NP separation, purification, and creation (145). **Table 3** shows several recently issued patents for nanoparticle formulations comprising natural anti-cancer molecules.

**Table 3:** Summary of recently published patents related to nanoparticle formulation with the natural anti-cancer molecule.

Patent number/year	Title	Description of patent	Compound/herbal plant	Treatment of cancer	Reference
IN202241000705 (2022)	Lung cancer treatment using astragalus, cisplatin *and* vinorelbine	It describes the novel drug formulations of astragalus, cisplatin *and* vinorelbine for treating lung cancer	Astragalus, cisplatin *and* vinorelbine	Lung cancer *and* non -small cell carcinoma [NSCLC]	(244)
IN202111049427 (2021)	A novel herbal composition for anticancer activity	This invention relates to the herbal composition for cancer treatment where *cinnamon zeylanticum J.presl.*, is utilized	*Cinnamomum zeylanicum J.presl.*	Used in different cancer treatments	(245)
IN202141046188 (2021)	Enhanced anticancer activity of quercetin-loaded TPGS(Tocopherol polyethylene glycol succinate) nanosuspension for drug imperious MCF-7(Michigan cancer foundation -7) human breast cancer cells	This study provides a novel insight into the mechanism of action of quercetin-induced apoptosis in human breast cancer cells	Quercetin	Human breast cancer	(246)
IN202021048696 (2020)	Cytotoxic herbal silver nanoparticles as a remedy for mammary carcinoma	The present invention is herbal extract mediated silver nanoparticles acting as a cytotoxic agent to mammary carcinomatous cells by showing G2/M-phase cycle arrest	*Brassica oleracea L.*	Mammary carcinoma, cervical cancer, hepatocarcinoma cell	(247)
IN202041023550 (2020)	The non-invasive, novel polyherbal synergistic nanoformulation for the effective prevention and arrangement of human lung cancer	This formulation possesses anticancer, cytotoxic and wound healing properties for effectiveness in lung cancer	Tridax procumben L.,*curcuma longa L.*, and *trachyspermum amm(L.)Sprague.*	Lung cancer	(248)
IN202041025649 (2020)	The non-invasive novel polyherbal formulation for the prevention and management of gastric cancer, its preparation and uses thereof	The polyherbal formulation having nine ingredients has given very positive leads and evidence regarding its efficacy in the effective prevention and management of gastric cancer and also with extended scope of teat colon cancer also	*Plumbago zeylanica L., Zingiber officials Roscoe., Terminalia chebula Retz., Indigofera tinctoria L., Syzygium aromaticum (L)Merr.andL.M.perry., Tamarindus indica L., Piper nigrum L., Cissus quandragularis L., Trachyspermum ammi(L) Sprague.*	Gastric cancer *and* colon cancer.	(249)
US20170258929 (2017)	The method uses and combination composition in cancer treatment	The conjugate of GnRH and curcumin give along or in combination with 2’,2’-difluoro-2’-deoxycytidine in pancreatic cancer treatment	Curcumin and its analog	Pancreatic cancer	(250)
EP3144006 (2017)	The combination of chemotherapeutic agent and curcumin analog used in the treatment of glioblastoma	Liposomes containing curcumin eliminate the QT- prolongation to treat glioblastoma	Curcumin	Glioblastoma	(251)
US20170035701 (2017)	Preparation method and uses of stabilized high drug load of nanocarrier	Formation of the micellar core of active compound by encapsulating the active compound by nanocarrier of lipid shell	Curcumin, reservatrol, honokiol, and magnolol	Brain cancer, liver cancer, and skin cancer	(252)
US20170189343 (2017)	Drug carrier for tumor-specific drug delivery and uses of it	It provides a nano drug carrier for tumor treating drugs to depolymerize -polymerize the human ferritin	Various anticancer bioactive compounds	Various hematological Cancer	(253)
US20170224636 (2017)	Curcumin-sophorolipid complex.	The present invention relates to a curcumin acidic sophorolipid complex is enhances the bioavailability of curcumin and nano- encapsulated in acidic sophorolipid	Curcumin	Specifically for breast cancer	(254)
US20160287533 (2016)	Bioavailability enhancing curcumin composition, method, and uses	The formulation which increasing the curcumin properties and treats the uncontrolled cellular growth in the human cell	It included at least one curcumin, resveratrol, catching derivative	Cervical cancer and precancerous cervical lesion	(255)
US20160287706 (2016)	Left helical 3D cage-like structure of DNA drug carrier nanocage	L-DNA (left helical structure of DNA) nanocage (3D cage) has high efficiency of cellular delivery so it is very suitable to deliver an active herbal drug into the cell	Genistein	Various cancer	(256)
US20140369938 (2016)	Curcumin-coated magnetic nanoparticles for biomedical application	Curcumin and its derivatives are coated with ultra-small superparamagnetic nanoparticles of iron oxide directly without losing the therapeutic properties of curcumin	Curcumin	Breast cancer, lung cancer, pancreatic cancer	(257)
US20160263221 (2016)	Information of pharmaceutical formulation of controlled drug targeted delivery system	It provides a container having at least one herbal drug and adapted to release herbal compounds inside the cancer cell	Curcumin	Lung, breast, prostate, and colon cancer	(258)
W02016014337 (2016)	Nanoemulsion based drug delivery system	Drug delivery system forming a nanoemulsion for an anticancer agent	Curcumin	Colorectal cancer	(259)
US20150314006 (2015)	Particulate drug delivery methods.	It provides preparation of drug-polymer or oligomer conjugate which is useful in *in-vivo* drug delivery for therapeutic application	Genistein-like species which having one hydroxyl group and a thiol group	Prostate cancer	(260)

## Challenges

Medicinal plants may have a large number of active ingredients, some of which may be beneficial in therapy while others may be harmful (261). There is a lack of standard protocols and meta-analytic evidence for the use of herbal medications. The predominant role of herbal medication in cancer is to treat symptoms of neoplasm and strengthen the immune system (262). Herbal medicines are used as adjuvant therapy to treat side effects associated with chemotherapy but they have not been used alone to cure or prevent cancer. *In vitro* studies have been successfully performed for the treatment of cancer but in clinical trials, herbal medicine did not succeed much (32). Herbal medicines can be useful in the case of primary cancer but they cannot be useful to treat severe cases of cancer until yet (32). Satisfactory evidence regarding the administration of herbal medication through the parenteral route and proper guidance for the route of administration is not available. A target-based specific treatment for the cancerous cell is not commercially available in herbal therapy. The database of specific interactions of these medications along with other prescription drugs is deficient (263). Several compounds show structural similarity but verification of their structural relationship and function are not well defined (264). In a patient taking cancer treatment, herbal medicine may interfere and alter pharmacokinetic properties. Along with the above challenges, one more challenge is the clarity of the mechanism behind its pharmacological action. While conventional medicine has a proper monitoring system, Ayurvedic treatments do not have a sufficient monitoring system, data analysis, objective level, measuring of the drug, and tools of standardization (265). When herbal medication is used along with other chemotherapeutic agents, it can reduce the bioavailability of chemotherapeutic agents (169).

Some of the herbal medications do not just act on target cells, but also a wide range of normal cells. The limitation of herbal medication is that it can have numerous targets. In some instances, herbal extracts may produce adverse effects. Resistance is common in herbal treatments such as camptothecin (266). Vincristine, a component of the vinca alkaloid, may cause swelling because of fluid accumulation in tissue. If a person consumes vinca, it may cause heart and blood vessel problems. Coma, drowsiness, nerve discomfort, motor dysfunction, and other neurological problems might occur because of vinca ingestion. Vinca alkaloids cause skin responses, as well as baldness and rapid hair loss. Indigestion, vomiting, and cramping are some of the gastrointestinal complications associated with this therapy (267). Decreased bone marrow activity and pain due to nerve damage are consequences associated with taxols (e.g., paclitaxel) (268). Chaga mushroom powder, which is used to treat cancer, has been linked to calcium oxalate buildup, which can lead to tubular damage in the lumin of tubules, and oxalate crystals may be observed (269). Treatment with glycyrrhiza may result in tumor-bearing pressure, lack of appetite, and a reduction in potassium levels in the blood. It also causes hypokalemic conditions when other hypokalemic drugs are co-administered with it (270, 271). Patients who choose herbal medication as a cancer treatment may have various safety problems, such as a reduction of cancer drug concentration in the blood. Mephitic symptoms have been observed in cancer patients who are receiving herbal medicines. Herbal medications, in addition to their benefits, have certain disadvantages, such as changes in metabolism caused by drug interactions and changes in absorption. The maidenhair tree increases the harmful effects of paclitaxel by blocking its metabolism (272). Menorrhagia and dementia can occur when herbal medications are taken in conjunction with pregnancy-preventive medications (273).

## Future Prospects

Currently, people are focusing on herbal medication, which is cheaper and is commonly available so, advancements in their formulation and proper drug delivery system should be introduced. Drug regulatory standards related to safety, quality, and efficacy should be developed and managed. Interaction of other anticancer drugs with herbal medications should be studied and data should be collected for proper buildup and usage of herbal drugs. A wide variety of compounds will be available because many types of research are ongoing for herbal medications. Advancement can be made by modification of constituents to produce semisynthetic medicines. Many genetically modified natural products can be established by the use of the latest biotechnological methods. Complementary and alternative medicine (CAM) formulations play an important role and have achieved significant results in anticancer treatment. Production, sale, marketing, and use of these medicines should be properly controlled by the government and specific norms should be introduced for their use. It has a high potential for treating cancer patients in the future. Some herbal drugs are administered *via* NPs-based formulations, which can cause toxicity, and some of their interactions should not be overlooked. As a result, a herbal bioactive chemical is loaded with a naturally produced NP, such as exosomes, which can create a high reaction while being low in toxicity. Many drugs are difficult to isolate at times, and the amount of substance available is sometimes limited. To solve this challenge, one must first obtain that specific gene implanted in a host and then provide the right environmental conditions for proper development and culture.

Theranostics is a concept that combines the phrases therapeutics and diagnostics, and it refers to the use of medications for cancer diagnosis and therapy using the concept of theranostic and for overcoming chemoresistance in breast cancer, the co-delivery system for doxorubicin (DOX) and *curcumin* (CUR) was designed, in a sustained pH-dependent manner, which acted as a chemosensitizer. The surface of magnetic NC was decorated by hydroxyapatite that was cross-linked with β-cyclodextrin leading to the improvement in *curcumin* bioavailability. The therapeutic efficiency was established by the reduction in tumor size (274, 275). In another research, nano curcumin was combined with rare-earth-doped upconversion nanoparticles (UCNPs). Poly (lactic-co-glycolic acid) (PLGA) was used to promote sustained drug delivery and drug release, targeting, minimizing the non-specific consumption by undesirable tissues, and enhancing its aqueous solubility (276). *Curcumin*-incorporated PLGA nanoparticles have exhibited increased cellular uptake, induction of apoptosis, suppression of tumor cell proliferation, and improvement in bioavailability. The advantage of combining UCNP and nano *curcumin* lies in the upconversion of visible light leading to the excitement of photosensitizer and favoring the fluorescence resonance energy transfer (FRET) which can be used in the examination of *in vivo* interaction between bimolecular entities (276).

Berberine with etoposide *via* albumin nanoparticle was used in the Nano-in Nano approach. Co-encapsulation of BER was hypothesized to reduce the therapeutic dose of ETP, which would minimize its toxicity and overcome the problem of developing resistance and enhance its antitumor efficacy (275). When given in lung cancer-bearing mice, it leads to a decrease in vascular endothelial growth factor (VEGF) expression level, triggered caspase activation with tumor cell apoptosis, and enhanced antiangiogenic effect that might refer to synergistic topoisomerase II inhibition and reduction of multidrug resistance (MDR) effect in A549 cells (232, 275, 277). Thus, various plants possessing anticancer properties can be formulated with modifications to make them suitable for use as a theranostic leading to a substantial increase in the potency of anticancer drugs in the future.

## Conclusion

Conventional cancer therapies are sometimes restricted due to their low specificity, which can result in significant side effects and toxicity, as well as the likely creation of MDR phenotype. As a corollary, the necessity to precisely destroy cancer cells, overcome MDR, and boost the specificity of a medication using spatial, temporal, and dosage regulation of its release is driving the hunt for more effective anti-cancer therapy. Natural products are a key source for the development of innovative anti-cancer medicines that may be used both preventively and therapeutically. Phytochemicals at lower doses initiate adaptive response but as one increases the dose it produces acute autophagic response and apoptosis. There are several types of drug carriers available today, each with distinct and varied qualities that make them suitable for the treatment of cancers. After appropriate studies, it is now possible to create multipurpose nanocarriers that permit the slow and selective release of various drugs at specific tumor locations. *In vitro* and preclinical research have shown that NPs transporting and delivering both chemotherapeutic drugs and natural ingredients are extremely efficient for both curative and chemosensitizing reasons. Allopathic drugs have some challenges with them, thus diverse research is ongoing for herbal medication to improve their effectiveness and for acknowledgment of their side effects. Plant extracts appear to be less effective and efficient than refined extracted herbal components. The inclusion of mixed elements may lead to antagonistic activity or undesirable side effects, as well as a reduction in the therapeutic potency of the main substance. The herbal component’s anticancer activity should be recognized based on IC50 for cancer cell survival. Herbal medications with high IC50 values have little anti-cancer potency and so are not good options. Drug-drug interactions among herbal ingredients and other traditional FDA-approved drugs should be extensively explored to find the most effective additive mixtures. The importance of herbal medicines may increase in the future *via* such a nano-drug delivery system. Further research is requisite to address the safety issues related to these systems.

## Author Contributions

VC has prepared the backbone of the manuscript. VC and AP wrote the original draft of the manuscript with KM and SS. Z-SC, ZW, KH, AP, and VC refined the first draft. Z-SC and VC critically revise the manuscript for intellectually correct content. KH supports the project. All authors contributed to the article and approved the submitted version.

## Conflict of Interest

The authors declare that the research was conducted in the absence of any commercial or financial relationships that could be construed as a potential conflict of interest.

## Publisher’s Note

All claims expressed in this article are solely those of the authors and do not necessarily represent those of their affiliated organizations, or those of the publisher, the editors and the reviewers. Any product that may be evaluated in this article, or claim that may be made by its manufacturer, is not guaranteed or endorsed by the publisher.
